# Assessment of labially impacted canines traction mode with clear aligners *vs*. fixed appliance: A comparative study based on 3D finite element analysis

**DOI:** 10.3389/fbioe.2022.1004223

**Published:** 2022-10-05

**Authors:** Qian Xia, Yao He, Lurong Jia, Chunjuan Wang, Weixu Wang, Chao Wang, Jinlin Song, Yubo Fan

**Affiliations:** ^1^ Stomatological Hospital of Chongqing Medical University, Chongqing, China; ^2^ Chongqing Key Laboratory of Oral Diseases and Biomedical Sciences, Chongqing, China; ^3^ Chongqing Municipal Key Laboratory of Oral Biomedical Engineering of Higher Education, Chongqing, China; ^4^ Key Laboratory of Biomechanics and Mechanobiology, Ministry of Education, Beijing Advanced Innovation Center for Biomedical Engineering, School of Biological Science and Medical Engineering, School of Engineering Medicine, Beihang University, Beijing, China

**Keywords:** labially impacted canine, finite element analysis, 3D printed, biomechanics, clear aligner

## Abstract

**Purpose:** The objective of this study was to evaluate and compare the biomechanical differences between clear aligner and fixed appliance in the traction of labially impacted canines based on 3D finite element analysis.

**Methods:** A series of patient-oriented finite element models were constructed, including a maxillary dentition with a right labially canine, maxilla, periodontal ligaments, traction attachments, and clear aligners. The two most common clinical scenarios were investigated: Scenario A: impacted canine (distal) and Scenario B: impacted canine (mesial). For each clinical scenario, three traction models with clear aligners and one fixed appliance model were established.

**Results:** In all four models, the impacted canines exhibited similar initial displacement tendencies of mesially rotated in Scenario A and distally rotated in Scenario B, and with small differences in periodontal ligament stress magnitude. However, the sum of the periodontal ligament stresses of the anchorage teeth in the clear aligner mode was in the range of 56.28–76.21 kPa and in the fixed appliance mode was in the range of 6.61–7.22 kPa. The maximum value of initial displacement of the anchorage teeth in the clear aligner mode was in the range of 13.71–19.72 μm, while in the fixed appliance mode was 3.10–3.92 μm.

**Conclusion:** For impacted canines, clear aligner mode and fixed appliance mode have little difference in biomechanical effect. However, the anchorage teeth in the clear aligner mode endure higher stress and show a more pronounced displacement tendency. In addition, the biomechanical effects of different clear aligner traction models are various but not obvious.

## Introduction

Nowadays, orthodontic treatment can correct various degrees of malocclusion and achieve good aesthetic results, periodontal health and temporomandibular joint (TMJ) stability (Hichijo et al., 2021). However, the correction of impacted teeth is relatively difficult in all malocclusion types. The impacted teeth refer to the ones that cannot erupt into its normal position in the arch because of the obstruction by the jaw, adjacent teeth, or mucosal tissue. Among them, the maxillary canine is the most commonly impacted tooth (1–3%) except for the third molars (Grover and Lorton, 1985), which are predominantly labially impacted in the oriental population (Oliver et al., 1989). In the past, the most common treatment for impacted canine was extraction, but the loss of canine can have a great impact on the patient’s aesthetics, occlusion, and TMJ. With the advancement of orthodontic technology, we have been able to align the impacted canine into arch with orthodontic traction (Cavuoti et al., 2016). However, the treatment of impacted teeth remains a challenge, especially when we treat patients rejecting fixed appliances and mini-screws (Zhong et al., 2006).

Currently, the research on the traction method of the upper jaw impacted canine is mostly focused on the clinical usage of fixed orthodontics (Iancu Potrubacz et al., 2018; Lena Sezici et al., 2020). The Kilroy spring, a mechanical attachment that can generate continuous force, can successfully achieve the traction of palatally or labially impacted canine. It is frequently used for traction of impacted canines due to its adjustability of traction direction and force (Bowman and Carano, 2003). Previous studies in the impacted canine treatment showed that traction with the Piggyback NiTi archwire is an efficient and effective method of aligning impacted teeth whilst maintaining a predetermined arch form, which could produce minimum negative effects on anchor units (Sandler et al., 1999). At present, fixed orthodontic attachments can effectively complete the traction orthodontic cases of impacted teeth.

Since the beginning of the aligner orthodontics era, clear aligner therapy is gaining popularity among both orthodontists and patients for its advantage of esthetics, comfort, and better oral hygiene over the fixed appliance. Advances in digital scanning, computer simulation and 3D printed technology have made it possible to use these devices to treat different types of malocclusion. However, due to the limited number of relevant studies, it is unclear whether the clear aligner mode is a better option for the traction of the impacted canine, and the differences between the mode with clear aligners and the mode with fixed appliance.

Three-dimensional finite element analysis (FEA) is an efficient computer simulation technique that has been widely used to calculate stress and deformation on geometric entities subjected to external forces (Zeno et al., 2020). It has the advantages of high precision, good repeatability, visualization of analysis results and so on. In addition, it can show the biomechanics inside the relevant soft and hard tissues (Zhang et al., 2008), which cannot be achieved *in vivo* studies. Accordingly, it has been widely used in biomechanical analysis in the field of orthodontics (Kuang et al.; Jia et al., 2022). With FEA studies, highly realistic clinical simulations can be performed to provide references for orthodontic clinical applications (Jin et al., 2010).

Therefore, we carried out a finite-element study to compare and evaluate the differences between the traction mode with clear aligners and the traction mode with fixed appliance, and to assess the discrepancies between diverse traction models of invisible orthodontics. Through the finite element analysis method, high fidelity simulation models of clear aligners and fixed appliance traction mode were established and compared in two common labial impacted canine clinical scenarios. This biomechanical analysis provides a theoretical basis for the selection of traction methods for impacted canine.

## Materials and methods

### Acquisition of medical image data

A 23-year-old male patient from the Department of Orthodontics, Affiliated Stomatological Hospital of Chongqing Medical University, with a labially impacted maxillary right canine was selected. This study was approved by the ethical committee of Stomatological Hospital of Chongqing Medical University and the ethics number was (2020) 094. The dentition and maxillary bone of the patient were scanned by Cone Beam Computed Tomography (Kava, Biberach, Germany) to obtain DICOM (Digital Imaging and Communications in Medicine) data. The working parameters of the scanning device were as follows: tube voltage 120 kV, tube current 5 mA, and voxel size 0.4 mm.

Inclusion criteria for the study were as follows: 1) Complete development of the jaw, and presence of all teeth (with the exception of third molars); 2) Presence of unilateral maxillary labially impacted canine; 3) Healthy teeth, no history of root canal treatment, no large-scale fillings, and no restoration crowns or dental implants; 4) Periodontal and temporomandibular joints were basically normal.

Exclusion criteria: 1) The crown of the maxillary posterior teeth is too short, and the clinical crown height on the palatal side is less than 4 mm; 2) Patients with history of maxillary surgery, trauma and tumor; 3) Developmental deformities of the maxilla, such as severe asymmetry, cleft palate, etc. that affect the integrity and structure of the jaw; 4) Metabolic-related diseases, especially those affecting bone metabolism.

### The construction of orthodontic model

**FIGURE 1:**
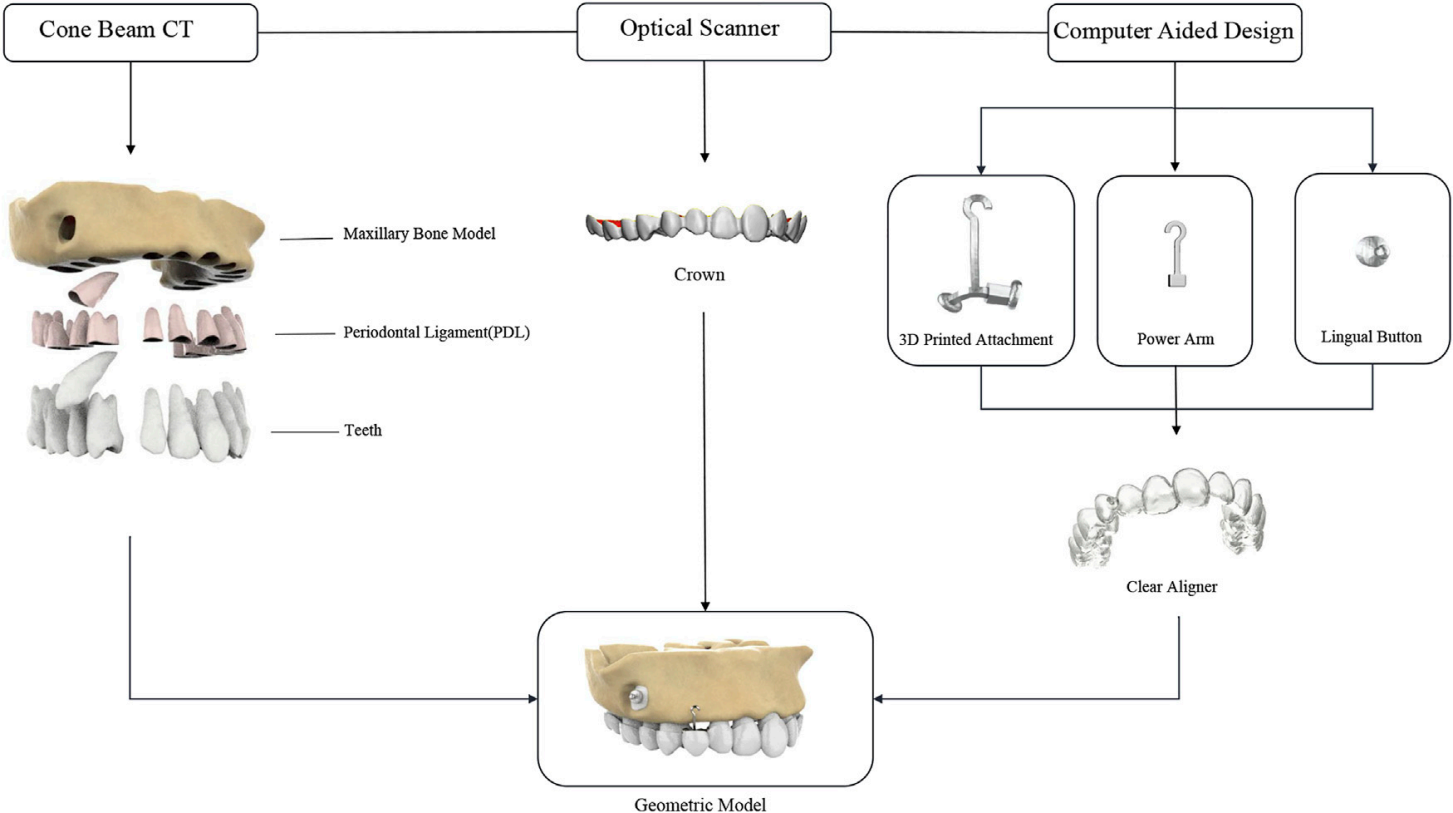
Computer-aided design model.

The DICOM data was imported into the Mimics system (version 17.0; Materialize, Belgium). The threshold range was adjusted based on the image’s gray level to segment the preliminary 3D models of the maxilla and dentition. The 3D model was exported in an STL file format. The STL file was imported into the Geomagic Studio software (version 2015; Geomagic, USA) to perform surface smoothing and fitting by using the relaxation command for the constructed models, which were then auto-surfaced to generate a computer-aided designs parametric model. Cortical bone, cancellous bone, and periodontal ligament (PDL) were obtained by scaling and Boolean operation, which were applied on individual teeth and maxilla surfaces. The average thicknesses of cortical bone and PDL were defined as 2.0 mm and 0.2 mm respectively (Jia et al., 2022), and cancellous bone was regarded as the residual. Finally, 3D geometric models of the maxilla, PDL, and teeth were created (Figure 1).

**FIGURE 2:**
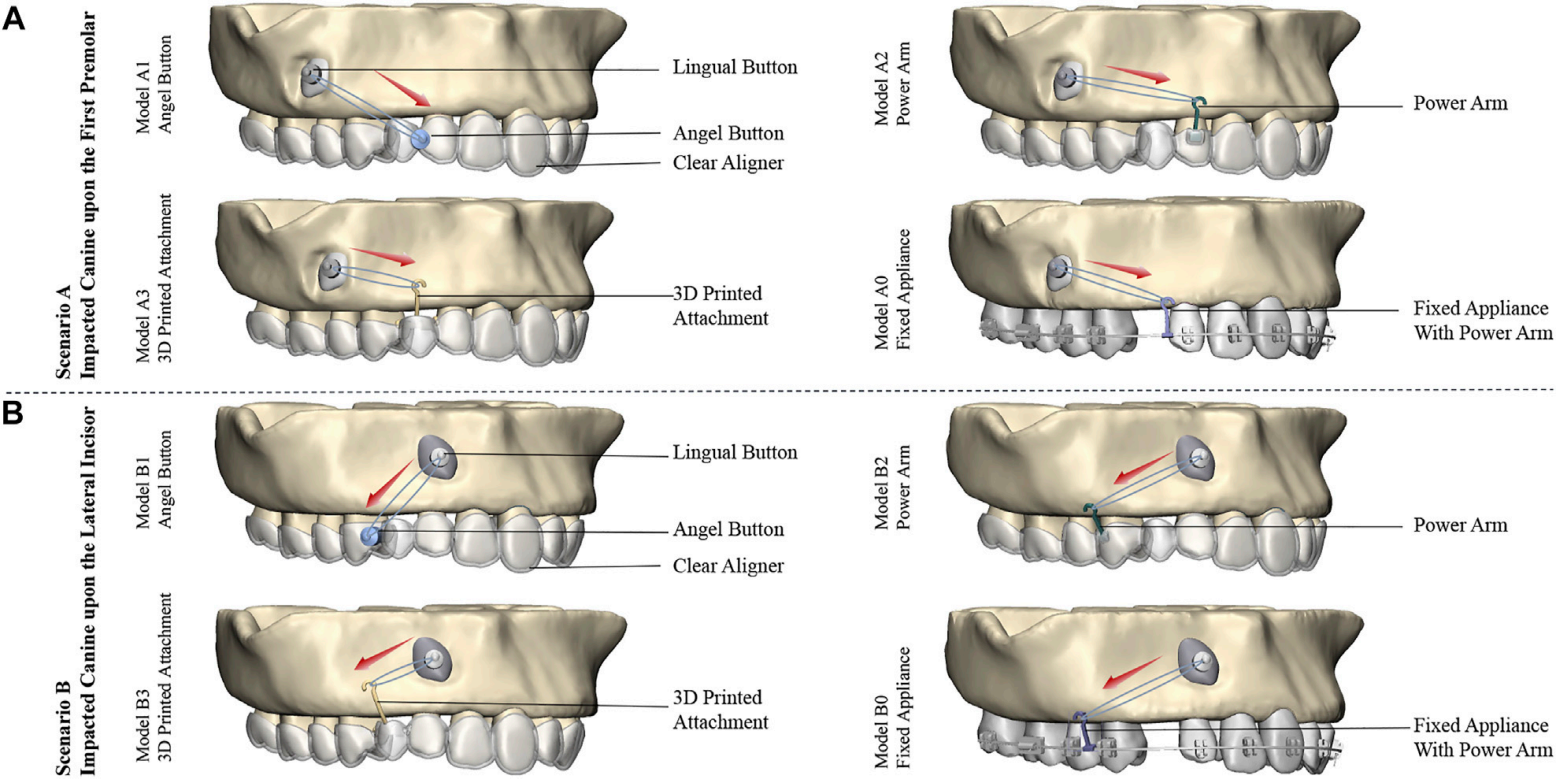
Traction models of the right labially impacted canines with clear aligners and fixed appliance: **(A)** Traction models of impacted canine in Scenario A. The clear aligner models are Model A1 (Angel Button), Model A2 (Power Arm), and Model A3 (3D Printed Attachment), and the control model is Model A0 (Fixed Appliance). **(B)** Traction models of impacted canine in Scenario B. The clear aligner models are Model B1 (Angel Button), Model B2 (Power Arm), and Model B3 (Printed Attachment), and the control model is Model B0 (Fixed Appliance). 8 models were designed with the same traction force of 0.6N. Red arrows indicate the direction of the traction.

As shown in Figure 2, three kinds of traction models with clear aligners (including the Angel Button model, Power Arm model, and 3D Printed Attachment model) and one Fixed Appliance model were constructed. Angel Button model is a new traction system of Anglealign@, which can achieve multi-angle traction by an angel button integrated with clear aligners. In Power Arm model, the geometry of the power arm was designed based on the most commonly used clinical dimensions, and the length is 7 mm. 3D Printed Attachment model consists of horizontal and vertical rods. The horizontal rod size can be designed according to the size of the gap, and is divided into left and right ends. Each end of the horizontal rod formed a stable bond with the teeth through the bottom plate, and the bottom plate was designed as the undercut-modified adhesive surface (Kuang et al., 2021). The horizontal rod was a sleeve-like connection, and the design of the sliding connection allows the movement of one end of the tooth while pulling the impacted canine. The vertical length and position of the hook can be individually designed according to the specific situation. In the Fixed Appliance model, to better simulate the clinical scenario, teeth are aligned and loaded with a relatively stiff rectangular archwire (0.018 × 0.025inch) (Hirschhaut et al., 2021).

### Material properties and meshing

The models were assembled and imported into ABAQUS software (version 6.14; SIMULIA, France). Each study subject was assumed to be as continuously homogeneous, isotropic linear elastomers, and the linear or nonlinear elastic property of PDL did not affect long-term orthodontic tooth movement, so the PDL model was considered a linear elastic material for the best accuracy-computational ratio (Kojima and Fukui, 2012). The material properties of the components were taken from previous studies and were summarized in Table 1 (Wagner et al., 2002; Tang et al., 2010; Ammar et al., 2011; Sung et al., 2015; Hedayati and Shomali, 2016; Chen et al., 2019; Mehari Abraha et al., 2019). C3D10M element type was used for the meshing of the 3D models, also called a modified tetrahedral quadratic element, which was especially suitable for contact calculations. The approximate number of nodes and mesh were shown in Table 1.

**TABLE 1:** Material properties and number of nodes and elements of the components of the finite element model.

Component	Young’s modulus (MPa)	Poisson’s ratio	Nodes	Elements
Teeth	18600	0.31	164181–175794	91180–98108
PDL	0.68	0.48	137271–140711	69922–70936
Cortical bone	13700	0.3	403430–448190	232268–248266
Cancellous bone	1370	0.3	222563–246345	125999–135923
Clear aligner	816.31	0.3	145800–165389	76950–88149
Lingual button	114000	0.34	2146–5027	1121–2774
Power arm	200000	0.3	2096–2580	965–1203
3D printed attachment	235000	0.33	6500–6895	3130–3428
Archwire	200000	0.3	15547–15687	7460–7526
Bracket	210000	0.3	24946–27806	11385–12741

### Boundary constraints and contact conditions

The Maxilla was fully restrained so that no rotation or displacement could occur. The contact relationships between the cortical and cancellous bone, alveolar bone and PDL, teeth and PDL, lingual button and the impacted canine, power arm and corresponding teeth, fixed arm of the 3D printed attachment and corresponding teeth brackets and corresponding teeth were defined as bonded connections. For better comparative analysis, clear aligners and fixed appliances do not exert additional force other than the traction force of the impacted canine. Accordingly, a tie constraint was used to model the interaction between brackets and wire, negating any relative motion between their surfaces. The external surface of the crown and the internal surface of the aligners were defined as non-linear face-to-face contact. Surface-to-surface contact was used between the aligner surface and teeth and power arm surfaces with a Coulomb friction coefficient of 0.2(Gomez et al., 2015). Considering that the friction coefficient of metal is small, the friction coefficient between the two fixed arms of the 3D printed attachment in this study is 0.2 (Yi-Min et al., 2015; Wen-Chao et al., 2017). 8 models of the two most common clinical scenarios were studied: Scenario A, in which the impacted canine was located upon the first premolar from a CBCT of a patient, included the clear aligner models: Model A1 (Angel Button), Model A2 (Power Arm), Model A3 (3D Printed Attachment) and control model: Model A0 (Fixed Appliance). Scenario B, the impacted canine was located upon the lateral incisor, which is the most common location for labial impaction (Kim et al., 2017). Scenario B contained the clear aligner models: Model B1 (Angel Button), Model B2 (Power Arm), Model B3 (3D Printed Attachment) and control model: Model B0 (Fixed Appliance). A traction force of 0.6 N was applied in all investigated schemes (Bishara, 1992).

### Calculation and analysis

The nonlinear iterative calculation was carried out in ABAQUS software (version 6.14; SIMULIA, France), and the results were output. The distribution of Equivalent Stress (von Mises) in the PDL, the average von Mises stress resulting from the initial force application of the PDL, and the maxillary initial displacement of the teeth were analyzed.

## Results

### Comparison of the maximum comprehensive displacements of the impacted canine

**FIGURE 3:**
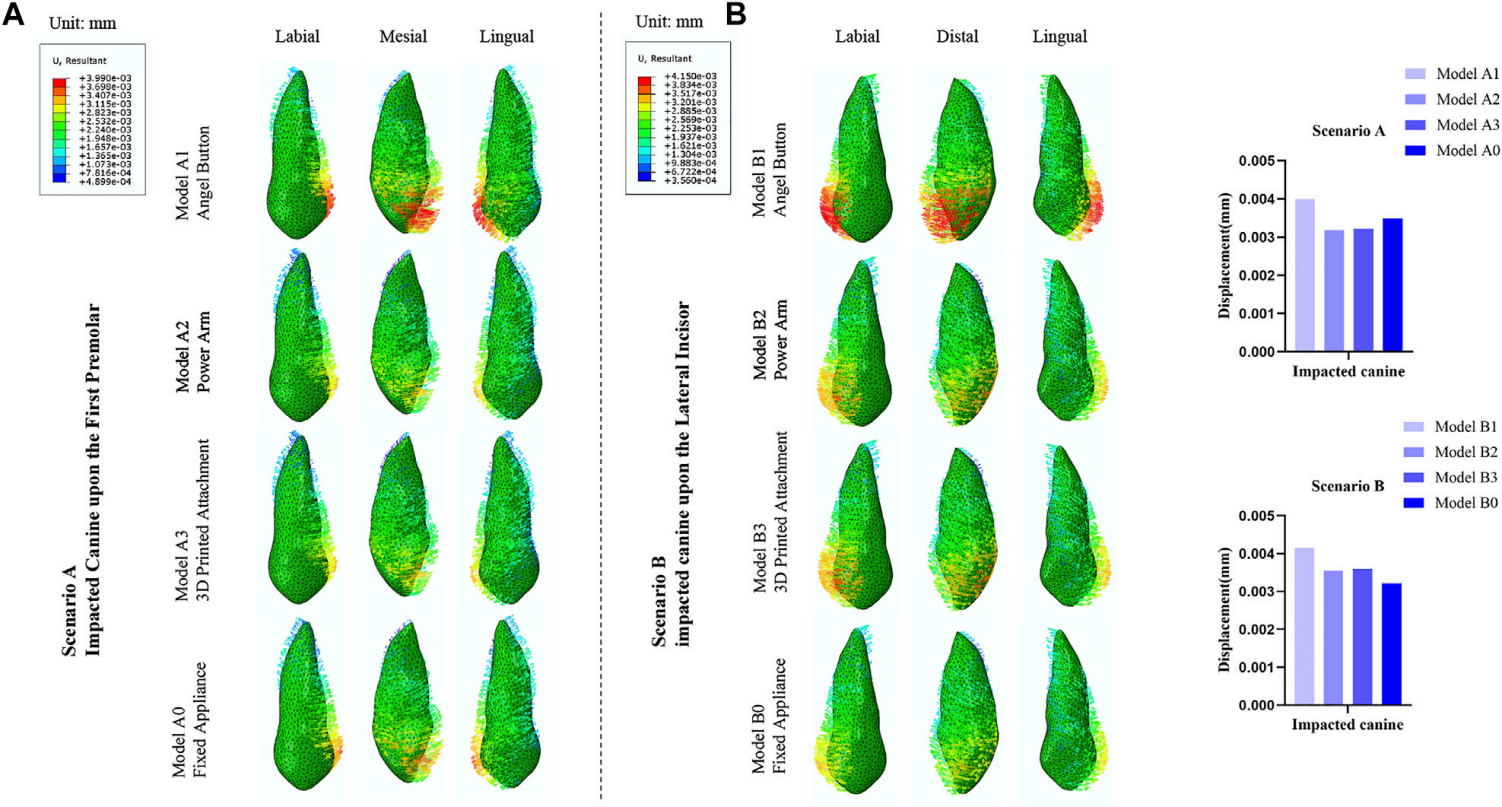
**(A)** Displacement tendencies of the impacted canine of Scenario A and Scenario B. **(B)** The maximum displacement of the impacted canine.

As displayed in Figure 3, in all models of the Scenario A and B, the displacement tendency of the crown and root of the impacted canine was opposite, and the maximum displacement was observed in the crown. Also, the impacted canine exhibited the similar tendency to mesially rotated. In Scenario A, the initial displacement of the impacted canine of Model A1, Model A2, Model A3 and Model A0 was 3.99 μm, 3.18 μm, 3.22 μm, and 3.49 μm, respectively. Impacted canines in Model A1 had the largest initial displacement, while the initial displacement of the impacted canine of Model A2, Model A3, and Model A0 were relatively close. In Scenario B, the initial displacement of the impacted canine of Model B1, Model B2, Model B3, and Model B0 was 4.15 μm, 3.55 μm, 3.60 μm, and 3.21 μm, respectively. The initial displacement of the impacted canine in the Model B1 was also greater than that in the other three models.

### Comparison of von mises stresses in the PDL of the impacted canine

**FIGURE 4:**
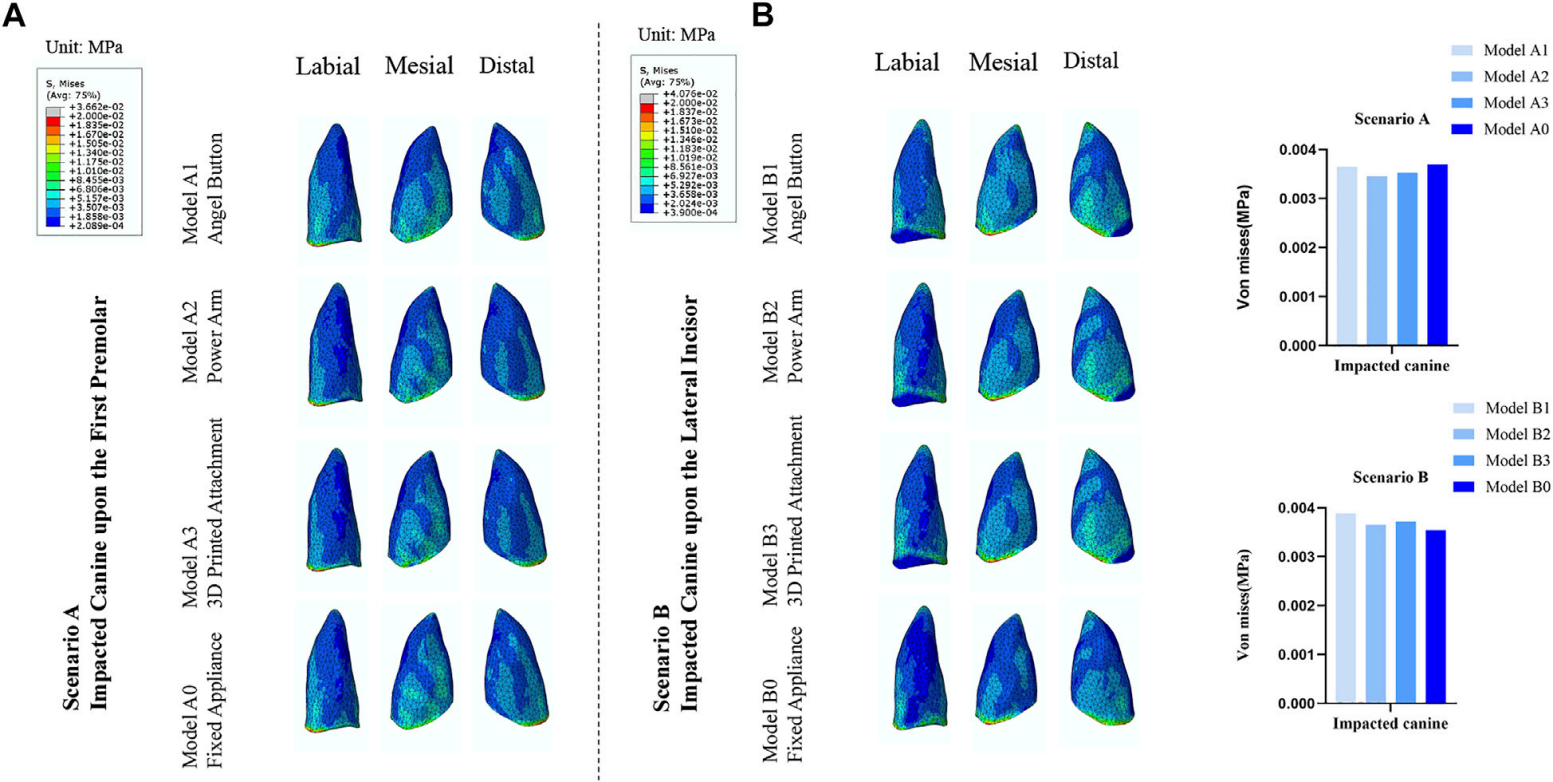
**(A)** The von Mises distribution (blue to gray reflects lower to higher stress) in the PDL of the impacted canine in response to the different traction models of Scenario A and Scenario B. **(B)** Stress value for average von Mises in the PDL of the impacted canine.

As shown in Figure 4, for the impacted canine in both scenarios, the stress was concentrated in the cervical area of the mesial and distal root surfaces, gradually decreasing towards the root tip. However, there was a small range of stress increases in the root tip area. In Scenario A, the stresses of the impacted canine PDL of Model A1, Model A2, Model A3 and Model A0 were 3.65 kPa, 3.46 kPa, 3.53 kPa, and 3.70 kPa, respectively. In Scenario B, the stresses of the impacted canine PDL of Model B1, Model B2, Model B3 and Model B0 in Scenario B were 3.89 kPa, 3.66 kPa, 3.72 kPa, and 3.55 kPa, respectively.

### Comparison of the maximum comprehensive displacements of the anchorage teeth

**FIGURE 5:**
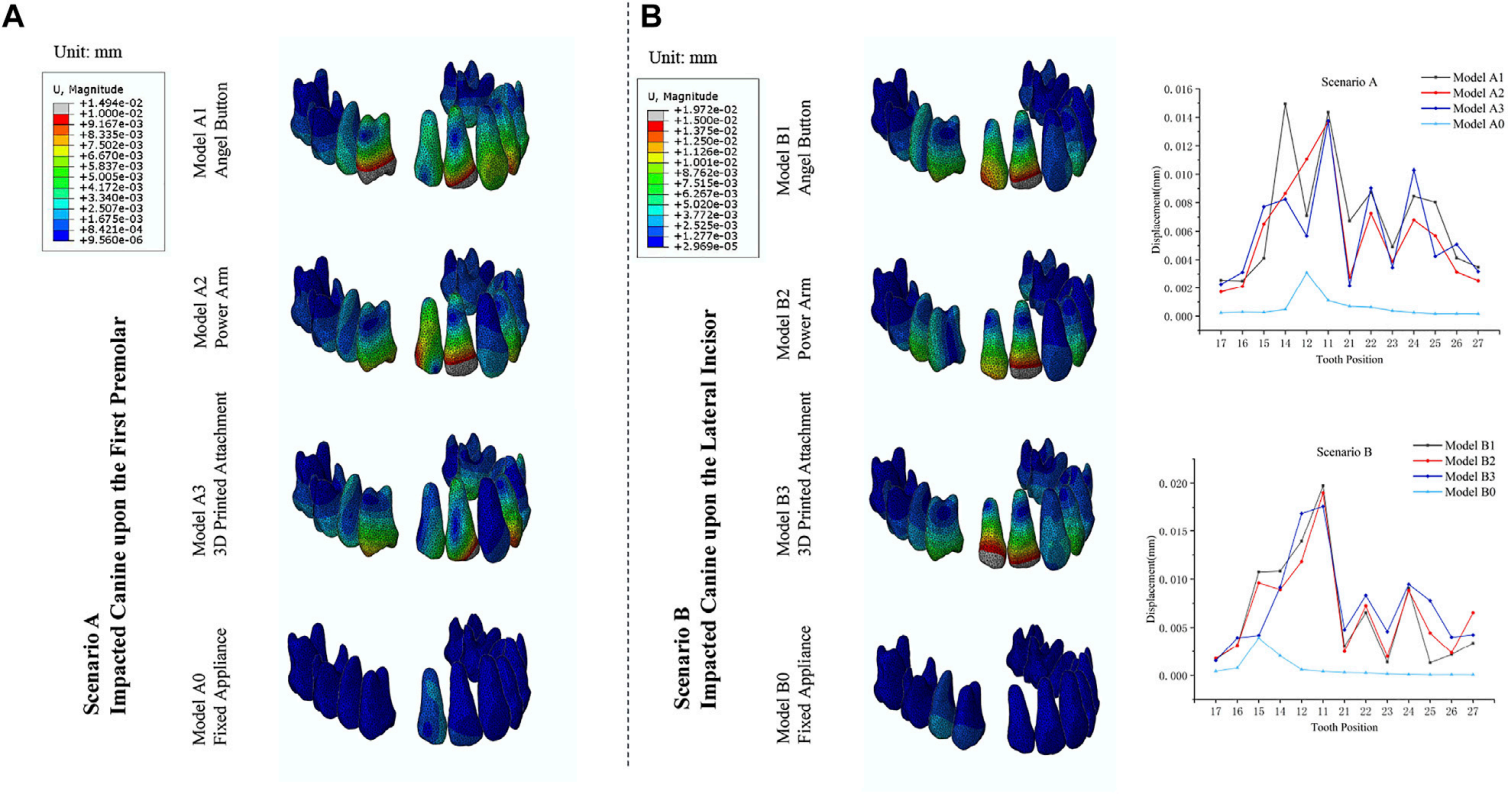
**(A)** Distribution of the initial displacements of the anchorage teeth in the traction models. **(B)** The maximum displacement of the anchorage teeth.

As depicted in Figure 5, overall, the value of initial displacement of the traction mode with clear aligners was significantly greater than that of the traction mode with fixed appliance. The max value of the initial displacement of the traction mode with fixed appliances was observed in the tooth position adjacent to the impacted tooth, and gradually decreased from the adjacent tooth toward the distal ends of the dentition. Relative to the traction mode with clear aligners, the max initial displacement was also observed for the teeth adjacent to the impacted tooth, and the initial displacement value of the anchorage tooth fluctuated up and down in an overall decreasing trend from the adjacent teeth of the impacted tooth to the distal dentition. For the traction mode with fixed appliance, the max value of Model A0 was observed at the adjacent lateral incisor (3.10 μm), and of Model B0 was observed at the adjacent second premolar (3.92 μm). For the traction mode with clear aligners, the max value of initial displacement of Model A1, Model A2, and Model A3 was observed at the adjacent first premolar (14.94 μm), central incisor (13.71 μm), central incisor (13.74 μm), respectively. The max value of initial displacement of Model B1, Model B2, and Model B3 was observed at the adjacent central incisor, and were 19.72 μm, 18.99 μm, and 17.59 μm, respectively.

### Comparison of von mises stresses in the PDL of the anchorage teeth

**FIGURE 6:**
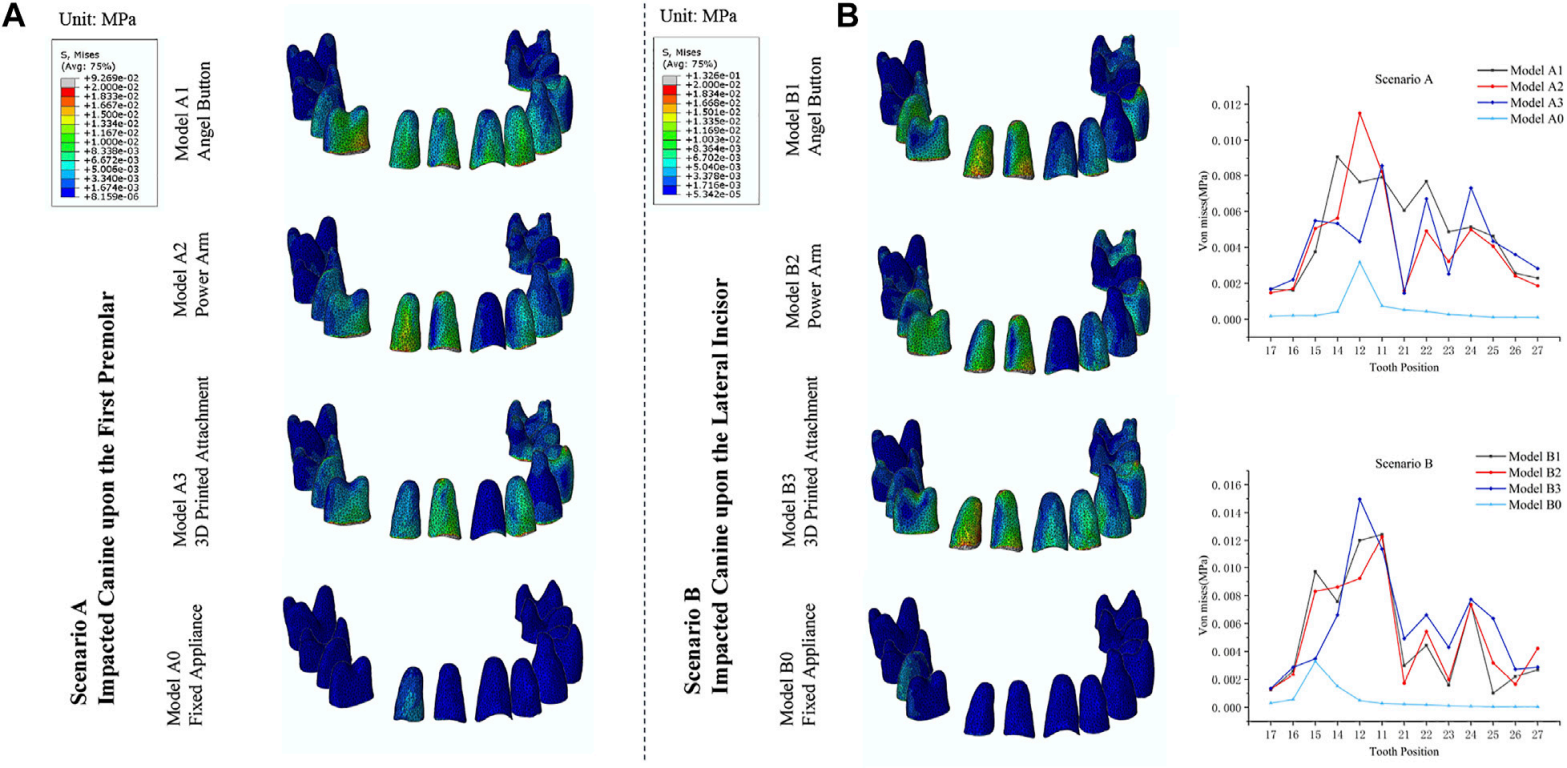
**(A)** The von Mises distribution in the PDL of the anchorage teeth in response to the different traction models used to move a maxillary right labially impacted canine of Scenario A and Scenario B. **(B)** Stress value for average von Mises in the PDL of the anchorage teeth.

As depicted in Figure 6, the stress distribution patterns on the anchorage teeth were similar to the distribution pattern of the initial displacement. For the traction mode with fixed appliance, the max value of the stress of Model A0 was observed at the adjacent lateral incisor (3.17 kPa) and Model B0 was observed at the adjacent second premolar (3.29 kPa). For the traction mode with clear aligners, the adjacent teeth of impacted canine also endured most of the stress. For Model A1, Model A2, and Model A3 the max value of stress was observed at the adjacent first premolar (9.07 kPa), lateral incisor (11.51 kPa), and central incisor (8.58 kPa), respectively. For Model B1, Model B2, and Model B3, the max value of stresses was observed at the adjacent central incisor 12.43 kPa, central incisor 12.27 kPa, and lateral incisor 14.96 kPa, respectively. Moreover, as shown in Table 2 and Table 3, for Scenario A and B, in the clear aligner mode, the total stresses on the anchorage teeth were about 8–11 times that of the fixed appliance mode. For Scenario A, among the traction mode with the clear aligners, the total stress value of the model A3 was slightly less than that of the other two models, and the stress value of its adjacent lateral incisors was 4.32 kPa, which was significantly smaller than that of Model A1 (7.63 kPa) and Model A2 (11.51 kPa). The sum of the stress value of the adjacent central incisor, lateral incisor and first premolar in Model A1, Model A2, and Model A3 were 24.59kPa, 25.33kPa, and 18.23kPa, respectively. For Scenario B, the adjacent lateral incisor in Model B3 endured nearly 20% (14.96 kPa) of stress, while the adjacent lateral incisor of Model B1 and Model B2 endured 17.96% (12.00 kPa), 13.64% (9.22 kPa), respectively. In this scenario, the total stress value of all anchorage teeth in Model B2 was less than the other two models. In addition, the sum of the stress value of the adjacent central incisor, lateral incisor, and first premolar in Model B2 was 30.10 kPa (44.52%), less than the 32.00 kPa (47.17%) in Model B1 and 32.97 kPa (43.27%) in Model B3 (Table 3).

**TABLE 2:** Von mises stress (kPa) on PDL of anchorage teeth and corresponding percentages with different traction models used to move a maxillary right labially impacted canine located upon the first premolar.

Model	17	16	15	14	12	11	21	22	23	24	25	26	27	Total
A1	1.67	1.61	**3.75**	**9.07**	**7.63**	**7.89**	6.05	7.66	4.86	5.13	4.62	2.56	2.28	64.78
	2.46%	3.00%	6.42%	14.56%	12.75%	12.85%	2.46%	12.72%	8.37%	8.96%	7.77%	4.18%	3.70%	
A2	1.47	1.70	**5.04**	**5.62**	**11.51**	**8.20**	1.58	4.90	3.21	4.99	4.06	2.41	1.86	56.55
	2.74%	3.18%	8.92%	9.67%	20.48%	14.44%	2.84%	8.79%	5.79%	8.63%	6.98%	4.25%	3.28%	
A3	1.67	2.20	**5.48**	**5.33**	**4.32**	**8.58**	1.45	6.68	2.51	7.30	4.34	3.60	2.82	56.28
	3.00%	4.15%	9.50%	9.10%	7.75%	14.91%	2.57%	11.87%	4.78%	12.56%	7.70%	6.34%	5.77%	
A0	0.17	0.20	0.20	**0.41**	**3.17**	**0.74**	0.52	0.43	0.26	0.19	0.11	0.11	0.10	6.61
	2.56%	3.03%	3.06%	6.33%	47.93%	11.15%	7.84%	6.48%	3.92%	2.86%	1.67%	1.63%	1.54%	

Numbers in bold type indicate higher stresses on PDL of adjacent teeth under specific model.

**TABLE 3:** Von mises stress (kPa) on PDL of anchorage teeth and corresponding percentages with different traction models used to move a maxillary right labially impacted canine located upon the lateral incisor.

Model	17	16	15	14	12	11	21	22	23	24	25	26	27	Total
B1	1.26	2.58	**9.71**	**7.57**	**12.00**	**12.43**	2.99	4.43	1.59	7.37	1.02	2.21	2.69	67.85
	1.86%	3.80%	14.31%	11.16%	17.69%	18.32%	4.41%	6.53%	2.34%	10.86%	1.50%	3.26%	3.96%	
B2	1.32	2.32	**8.32**	**8.61**	**9.22**	**12.27**	1.73	5.43	1.99	7.33	3.18	1.66	4.23	67.61
	1.95%	3.43%	12.31%	12.73%	13.64%	18.15%	2.56%	8.03%	2.94%	10.84%	4.70%	2.46%	6.26%	
B3	1.35	2.87	**3.48**	**6.62**	**14.96**	**11.39**	4.92	6.62	4.30	7.73	6.37	2.72	2.88	76.21
	1.77%	3.77%	4.57%	8.69%	19.63%	14.95%	6.46%	8.69%	5.64%	10.14%	8.36%	3.57%	3.78%	
B0	0.31	0.57	**3.29**	**1.51**	**0.50**	0.28	0.23	0.19	0.11	0.08	0.05	0.05	0.05	7.22
	4.29%	7.89%	45.57%	20.91%	6.93%	3.88%	3.19%	2.63%	1.52%	1.11%	0.69%	0.69%	0.69%	

Numbers in bold type indicate higher stresses on PDL of adjacent teeth under specific model.

### Comparison of the maximum comprehensive displacements of the adjacent teeth

**FIGURE 7:**
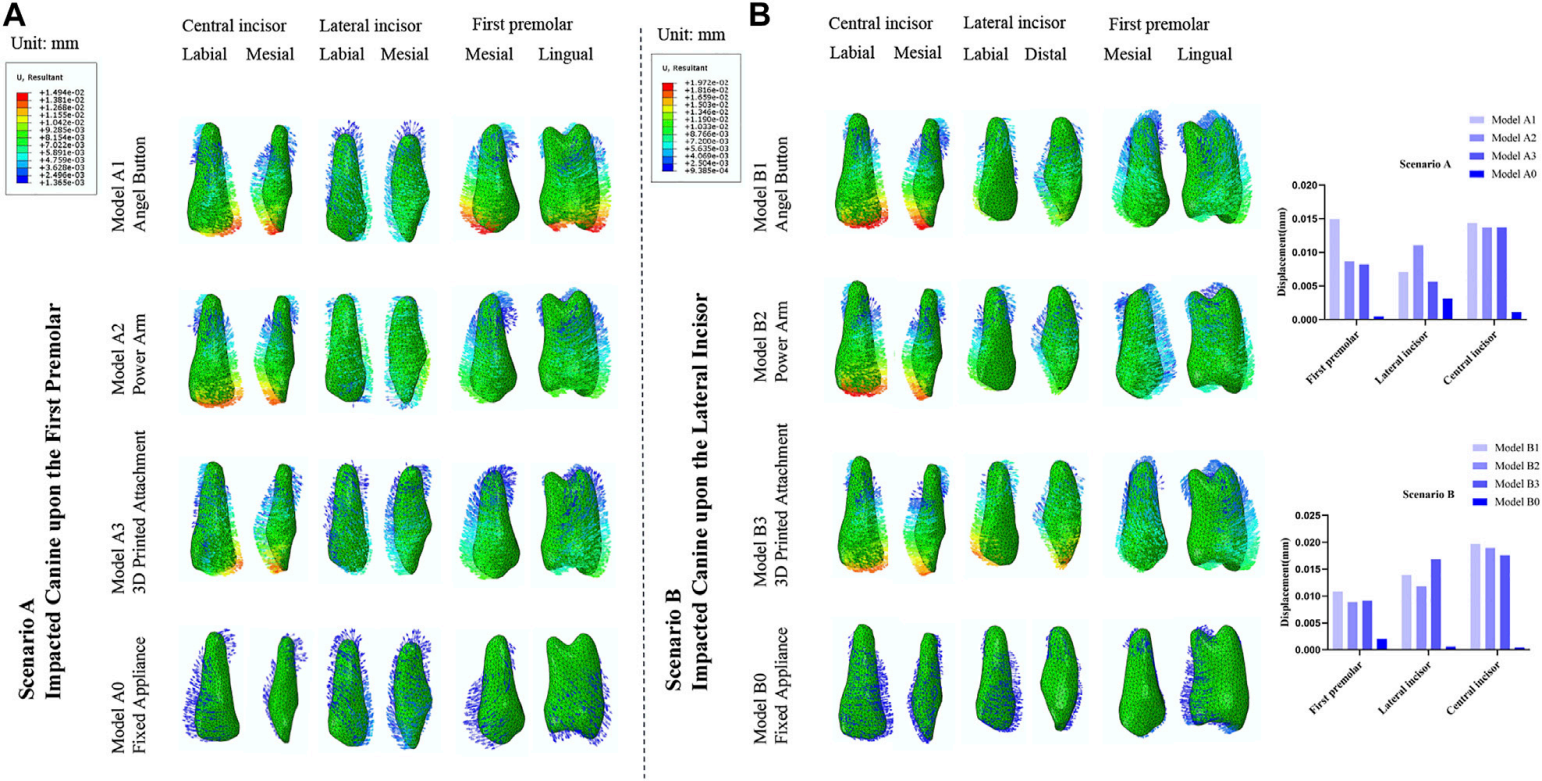
**(A)** Displacement tendencies of the adjacent central incisor, lateral incisor, and first premolar in the traction models of Scenario A and Scenario B. **(B)** The maximum displacement of the adjacent central incisor, lateral incisor and first premolar.

As displayed in Figure 7, for Scenario A and B, the initial displacement of the adjacent teeth in the clear aligner mode was significantly greater than in the fixed appliance model (Figure 7B). The central incisors in the three traction models with clear aligners were mesially tipped, while the first premolars were distally tipped and intruded. For Scenario A, the lateral incisor of Model A1 was mesially rotated and intruded, while the lateral incisor of Model A2 and Model A3 were distally tipped and intruded. Three teeth adjacent to the impacted tooth tended to be intruded. Relative to clear aligner models, the lateral incisor in fixed appliance models was distally rotated and intruded, and the central incisor and the first premolar were both distally tipped. For the four models in Scenario B, the central incisors exhibited a similar displacement trend of mesially tipped inclination, and the lateral incisors showed a similar displacement trend of distally tipped inclination. The first premolar of Model B1 was distally tipped and intruded, the first premolar of Model B2 was mesially tipped and intruded, and the first premolar of Model B3 was distal-labially tipped. The first premolar of the fixed appliance was lingually tipped and extrusion, while the central incisor and the lateral incisor were mesially tipped. Compared with the fixed appliance mode, the anchorage teeth of the clear aligner mode showed more complex and uncontrollable displacement tendencies, especially for the adjacent teeth (Figure 5B).

### Comparison of von mises stresses in the PDL of the adjacent teeth

**FIGURE 8:**
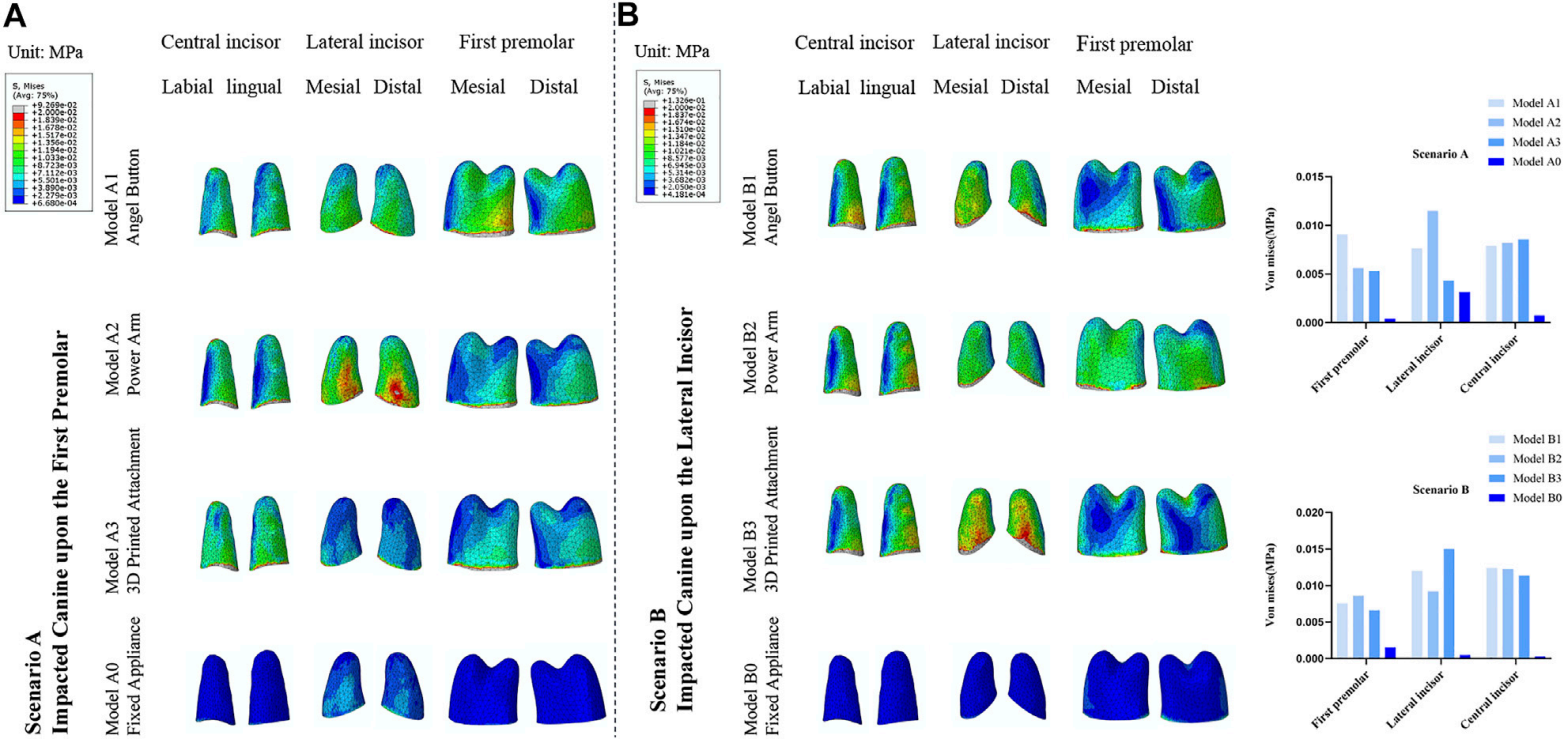
**(A)** Distribution of von Mises stresses in the PDL of the adjacent central incisor, lateral incisor, and first premolar in the traction models of Scenario A and Scenario B. **(B)** Stress value for average von Mises in the PDL of the adjacent central incisor, lateral incisor, and first premolar.

As displayed in Figure 8, for the four models in Scenario A, the stress of PDL of the adjacent lateral incisor and first premolar was mainly distributed in the cervical of the mesial and distal of the root surface and gradually decreased toward the apex. The stress of the PDL of the central incisor was concentrated on the labial and lingual cervical of the root surface. For Scenario B, the stress of PDL of the adjacent central incisor and lateral incisor was mainly distributed in the cervical of the mesial and distal surface of the root in all four models. However, the distribution of PDL stress of adjacent first premolar was different. The stress of PDL of the adjacent first premolar of Model B1 and Model B2 was mainly distributed in the cervical of the mesial and distal surface of the root, while of the Model B3 and Model B0 was mainly distributed in the cervical of the labial and lingual surface of the root.

## Discussion

By simulating the traction process of the impacted canine under different traction modes, the biomechanical differences between clear aligners and fixed appliances in the traction of labially impacted canines were evaluated in this study. The differences between diverse traction modes of clear aligners were also compared. Based on the results of this study, we found that under the traction of the clear aligner mode and the fixed appliance mode, the impacted canines exhibited similar initial displacement tendencies, and have similar distribution of PDL stress. However, for the anchorage teeth, traction mode with the clear aligners will cause larger PDL stress and bring more obvious and uncontrollable displacement compared with fixed appliance mode, especially for adjacent teeth. In addition, the biomechanical effects of different clear aligner models are different but not obvious. For the impacted canine located upon the first premolars, the 3D Printed Attachment model has the least effect on adjacent incisors, and for the impacted canine located upon the lateral incisor, the Power Arm model has the least effect on adjacent incisors. Thus, the basic goal of this study to provide critical information for selecting the most appropriate appliance in clinical practice has been accomplished.

According to previous studies conducted by scholars on visiting patients, it was found that the prevalence of impacted teeth in orthodontic clinics was high, especially the maxillary canines. (Fardi et al., 2011; Deng et al., 2012). In orthodontic clinics, impacted teeth are increasingly becoming one of the focuses of orthodontists. Different methods have been invented to pull the impacted canine to its desired position. These methods include the K-9 spring (Shastri et al., 2014), the ballista spring (Jacoby, 1979), the cantilever spring (Nakandakari et al., 2016), elastomeric chains (Yadav et al., 2011) or threads, and piggyback (double archwire) (Sandler et al., 1999). Indeed, the traction phase of the impacted canines used to be considered possible only by the traction method with fixed appliances (Kornhauser et al., 1996; Sander et al., 2006) or min-implants (De Clerck et al., 2002; Heravi et al., 2014, 2016). However, compared with the fixed appliance and mini-implants, the traction method with clear aligners has the advantages of being more aesthetically pleasing, clean, and non-invasive. Also, the traction of the impacted canine can be carried out early, without waiting until the arch was aligned, any existing crowding was resolved, and sufficient space was provided for the canine, which can save correction time. Accordingly, this study selected three invisible orthodontic traction methods, and compared them with traditional fixed orthodontic methods through three-dimensional finite element analysis. From the perspective of biomechanics, evaluate and compare the differences between clear aligner mode and fixed appliance mode, and assess the discrepancies between diverse traction models of invisible orthodontics.

Based on the same traction force and similar traction direction, the effects of the clear aligner mode and the fixed appliance mode on the impacted canine were similar. Among the four models, the impacted canine exhibited similar initial displacement tendencies, and with small differences in periodontal ligament stress magnitude. Moreover, the PDL stress distribution was similar, implying that all three clear aligner models had similar traction effects on the affected canines as the fixed appliance models. Similar to the traction mode with fixed appliance, in the clear aligner mode, it was still the first and second adjacent teeth to the impacted canine that endured most of the stresses (Zeno et al., 2019), and the stresses on the adjacent first premolar, lateral incisors, and central incisors account for 30–50% of the total stresses (Tables 2, 3). These findings are consistent with previous studies, which makes our research results more reliable. More importantly, it reminds us that in clinical practice, the state of adjacent teeth should be highly concerned, whether in fixed appliance mode or in clear aligner mode.

The stress distribution and displacement of the anchorage teeth are also important considerations during traction in impacted canines. Poor traction methods may have adverse effects on the anchorage teeth, such as undesirable tooth movement and root resorption. Although the initial force magnitude of these models was the same, the biomechanical response of the anchorage teeth in clear aligner mode and fixed appliance mode was not the same. The different responses on the anchorage teeth may be the result of different anchorage systems caused by different traction patterns.

In the clear aligner mode and the fixed appliance mode, the sum of the PDL stress values of the anchorage teeth was different, and the PDL stress distribution was also different. The results of this study showed the sum of the PDL stresses of the anchorage teeth corresponding to the traction mode with fixed appliance was 6.61–7.22 kPa and the value was consistent with the study by Zeno et al. (Zeno et al., 2019). However, the anchorage teeth of the clear aligner mode were subjected to 8–11 times the stress of the fixed appliance mode. The fixed appliance can better bind the entire dentition together through the archwire and brackets, which can form more powerful support. However, in contrast to fixed appliances, the inadequate stiffness of aligner materials and its frictional contact with the teeth makes it hard to sustain the original shape and form a stable and strong anchorage. From the results (Figure 6), it can be found that the PDL stress of the anchorage teeth in the clear aligner models fluctuated from the traction point to the distal end of the dentition, which was related to the complex action mode of the clear aligners. The stress on the PDL of the anchorage teeth is not only related to the distance of the traction point but also affected by factors such as the size of the contact area with the clear aligners.

In addition, the displacement of the anchorage teeth in the clear aligner mode and the fixed appliance mode was different. Compared with the fixed appliance mode, the application of the traction mode with the clear aligners yielded a more pronounced and uncontrollable displacement of the anchorage teeth, especially for adjacent teeth (Figures 5, 7). Although the movement of the teeth can be better controlled by adding attachments in clinical practice (Gomez et al., 2015), the effect is limited and the occurrence of greater stress cannot be avoided (Barone et al., 2017). In terms of biomechanics, the fixed appliance mode had more advantages than the clear aligner mode in both scenarios. However, in different scenarios, the clear aligner traction models that had advantages was different.

The difference between the three clear aligner traction models was mainly reflected in the impact on adjacent teeth. Other than being immediately adjacent teeth, lateral and central incisors have smaller root surfaces area than premolars and molars, and this anatomical feature can lead to a greater risk of root resorption. Thus, more attention should be paid to the adjacent incisors. According to the results of this study, in Scenario A, Model A3 had the least effect on adjacent lateral incisor, and the total amount of stress of the adjacent first premolar, lateral incisor, and central incisor was comparatively lower than the other clear aligner traction models. Also, the total stress of the anchorage teeth of Model A3 was slightly less than the other two models. However, in Scenario B, the stress on the lateral incisors in Model B3 increased due to the change in the direction of traction and was significantly greater than the stress on the lateral incisors in the other two models. Model B2 had the least effect on the adjacent lateral incisor for the reason that the main action site of the Power Arm model was located on the first premolar. Moreover, in Model B2, the total stress of adjacent first premolar, lateral incisor, and central incisor were relatively lower than that of the other two models.

The more pronounced and uncontrollable movement of the anchorage teeth can have side effects on the teeth, occlusions, and TMJs. Root resorption is one of the common complications during orthodontic treatment, and it has been reported that 91% of teeth underwent some degrees of root resorption after orthodontic treatment (Lund et al., 2012). Excessive unnecessary movement increases the risk of root resorption (Segal et al., 2004; Lin et al., 2022). Meanwhile, excessive undesirable movement of the anchorage teeth aggravated the malocclusion status, which increased the treatment difficulty and duration. Conversely, prolonged treatment time bring extra risk for root resorption (Apajalahti and Peltola, 2007; Zhang et al., 2015). Occlusal abnormalities caused by excessive and uncontrollable movement, may lead to biomechanical environment changes of the TMJ, which probably related to TMJ disorders (Liu et al., 2021). Compared with the clear aligner mode, the stronger anchorage of the fixed appliance mode can bring lower movement of the anchorage teeth and decreased related side effects. However, a more rational design of the clear aligners sequence or more appropriate target position of teeth can overcome these potential biomechanical risks to some extent.

In order to achieve early traction, and make it possible for the orthodontist to continue working with aligners, we designed a 3D printed personalized attachment. However, we found that the anchorage of the 3D Printed Attachment traction model was still insufficient compared to the traditional fixed appliance traction model. Future research would attempt to design better individual attachments to form stronger anchorage systems, assisting clear aligners to tract the impacted teeth aesthetically, comfortably, and efficiently. Additionally, our comparative study lacks validation in clinical cases. At present, our research group is carefully selecting suitable cases for the corresponding comparative study, but it takes a long time. Clinically, appropriate traction points are set on a case-by-case basis, so our models may not reflect the specific data of individual cases and specific conditions. Even so, the quantifiable results offer an addition to the literature and reveal a tendency such as the higher stresses on the anchorage teeth were observed in the traction mode with the clear aligners compared to the traction mode with fixed appliances. Further research would likely explore these trends with additional conditions, such as different traction points, root length, aligner material property, and specific attachments.

## Conclusions

For impacted canines, clear aligner mode and fixed appliance mode have little difference in biomechanical effect.

For the anchorage teeth, traction mode with the clear aligners causes larger PDL stress and bring more obvious and uncontrollable displacement compared with fixed appliance mode, especially for adjacent teeth.

The biomechanical effects of different clear aligner models are various but not obvious. Adjacent incisors endure higher stress and show a more obvious undesirable displacement tendency. For the impacted canine located upon the first premolars, the 3D Printed Attachment model has the least effects, and for the impacted canine located upon the lateral incisor, the Power Arm model is more appropriate.

## Data Availability

The original contributions presented in the study are included in the article/Supplementary material, further inquiries can be directed to the corresponding authors.
